# Breast Cancer Awareness and Screening Among Menopaused Females in Al-Qassim Region

**DOI:** 10.7759/cureus.41680

**Published:** 2023-07-11

**Authors:** Saleh a AlSuwaydani, Abdulaziz S Alshamikh, Rayan A Alotaibi, Khalid O Almutairi, Buthaina h Alkhulifi

**Affiliations:** 1 Department of Surgery, Unaizah College of Medicine and Medical Sciences, Qassim University, Unaizah, SAU; 2 Department of Surgery, Unaizah College of Medicine and Medical Sciences, Qassim University, Unaziah, SAU

**Keywords:** ksa:kingdom of saudi arabia, alqassim region, awareness of breast cancer, post-menopausal women, mammography, screening, breast cancer

## Abstract

Background: Breast cancer (BC) is one of the most prevalent types of cancer among women worldwide including those in Saudi Arabia. The risk of developing BC can be lowered by reducing risk factors through early screening and by women having full knowledge of this condition. The aim of this study is thus to evaluate knowledge of the importance of early screening and detection of BC among post-menopausal women in Saudi Arabia’s Qassim region and to compare it with pre-menopausal women.

Methodology: A cross-sectional study was conducted among post-menopausal women in the Qassim region. Data were collected by using a pre-tested, pre-coded, validated self-administered online questionnaire. Data were analyzed using SPSS (Social Package of Statistical Science) Statistics version 23.0.

Results: Data were collected from 1386 women who agreed to participate in this study, of which 484 women reported that their menstruation had stopped (34.9%). In general, it was found that 73.7% of the participants had adequate knowledge with a significant difference between pre-menopausal and post-menopausal women. Concerning knowledge of BC (p = 0.042), pre-menopausal women had a higher level of knowledge (75.5% had adequate knowledge compared with 70.5% of post-menopausal women). Considering the source of knowledge of the participants regarding BC, websites or social media is considered the main source for 71.8% of the participants, followed by family and friends (52.2%). Concerning the knowledge about the risk factors of BC, 26.4% of the participants reported that they did not know them, and 11.8% of the participants did not know any of the symptoms of BC.

Conclusion: In this study, the knowledge of post-menopausal women was found to be adequate; however, it is significantly lower than that of the pre-menopausal women. Educational level is a significant factor that affects the level of knowledge regarding BC risk factors and different modalities for diagnosis and approaches for management, and this indicates the importance of increasing interest in education in our society.

## Introduction

Breast cancer (BC) is a progressive disease, with small tumors likely to develop at an early stage and early detection likely to result in successful treatment and a good prognosis [1-3]. BC is one of the most common types of cancer observed among women, and it is the second most common cancer overall after lung cancer [3,4]. In 2020, 2.3 million women worldwide were diagnosed with BC, with 27,885 diagnosed in the Kingdom of Saudi Arabia, compared to 82,640 in the preceding five years, i.e., before 2020, and over 675,000 fatalities due to BC were documented globally. By the end of 2020, 7.8 million women living had been diagnosed with BC in the previous five years, making it one of the most prevalent types of cancer in the world, and BC occurs in a large proportion and sees increasing rates in later life [5,6].

BC symptoms are divided into two categories: breast-related and non-breast-related. They can range in severity from moderate to severe, but it must be noted that not every patient will present symptoms in the same manner; they may vary from person to person. Painful or non-painful breast lump, nipple abnormalities such as discharge or bleeding, breast bruising, breast skin abnormalities, breast rash, breast ulceration, breast contour abnormalities, and breast pain are the most common breast-related symptoms, of which non-painful breast lump is the most common. There are also several non-breast-related symptoms, the most prevalent of which include an axillary lump, back discomfort, and weight loss [7]. Many risk factors increase the risk of developing BC among women, including family history of BC, past occurrence of BC in females, age, smoking, alcohol consumption, obesity, lack of exercise, and use of both hormone replacement therapy and birth control pills. Early menarche, late menopause, delay in having the first child, lack of breastfeeding, and radiation exposure are also considered risk factors [8].

BC in Saudi Arabian women is usually diagnosed in the advanced stage, and 14% of them experience death. However, it has been reported that an increased level of BC awareness and knowledge among women leads to early screening and detection, thereby increasing the chances of early treatment, better prognosis, and recovery [9]. Two hundred and thirty-two students and 68 faculty members of Najran University, Saudi Arabia, were included in a study done in 2020, which illustrated that 75.3% of subjects had an adequate level of general knowledge about BC, but it was observed that 94.3% of them had poor knowledge about the symptoms of BC [10]. L Linsell and his colleagues in England who assessed the awareness of BC among older women revealed that the majority of participants were aware that a lump is a symptom of BC, but they were not aware of non-lump symptoms. Further, around half of the participants believed that the chance of developing BC is less than one in 100 [11]. In the United Arab Emirates, a study that was conducted to evaluate BC screening awareness, knowledge, and practice has revealed the paucity of knowledge about BC screening. In addition, over half of the participants had never undergone a clinical breast examination or mammography, indicating a lack of awareness about BC screening techniques [12].

Al-Qassim region is a province in Saudi Arabia with a population of over one million people, 626,206 of whom are female. Although a few studies have been done on this topic, none have been conducted among post-menopausal women from the Al-Qassim region. Therefore, this research aims to highlight the importance of early screening and full knowledge of this disease in reducing the number of deaths, lowering the risk of developing BC, and improving the survival rate of post-menopausal women in the Qassim region, as the risk of developing BC can be reduced through early screening and women’s comprehensive knowledge of this disease.

## Materials and methods

Setting and participants****

This is an observational cross-sectional survey design based on an electronic interview survey. Interviewers used an electronic questionnaire built using Google Forms^TM^ and targeting women living in the Al-Qassim region, Saudi Arabia. Our sample size was calculated as follows. It initially included 392 women (based on an error of 5%, a confidence interval of 95%, a prevalence of 52.1% based on the previous data from Khadiga F. Dandash) [4] on knowledge, attitude, and practice surrounding BC and screening in female teachers of Buraidah. By adding 40 participants (10%) to compensate for non-responders and defaulters, the total minimum sample size was estimated to be around 432 women. The technique was a non-probability convenience sampling technique. 

Data collection methods

We used a questionnaire that was initially developed, pre-tested, and validated by Alsowiyan AA, Almotyri HM, Alolayan NS, Alissa LI, Almotyri BH, and AlSaigh SH [13]. The questionnaire is divided into four domains: the first domain includes the consent of participation, the second domain includes the socio-demographics data, menstrual history, and any history of BC, the third domain has 22 questions that asses the knowledge of BC and screening methods, which is divided into two sections, one with the general knowledge (total eight questions) and another one where we discuss many aspects of BC knowledge in detail such as symptoms and risk factors (remaining 14 questions). The last domain asks about the personal experience, which consists of seven questions. 

Data analysis plan

The assessment of overall knowledge was based on knowledge questionnaires that consisted of eight out of the 22 questions (general knowledge), wherein we identified the most appropriate answer for each question which we coded as 1 while the wrong answer was coded as 0. Based on the result, a score range of 0-8 had been generated. By using a cutoff point of 60%, poor knowledge was classified if the participants obtained a score of 4 points or less and good knowledge if participants obtained a score of more than 4 points.

Data were coded, entered, and analyzed using the SPSS (Statistical Package for Social Science) Statistics version 23.0 (IBM Corp., Armonk, NY). Qualitative data were expressed in the form of numbers and percentages (No. and %). A chi-square (χ^2^) test was used to examine the qualitative data between the two groups.

Ethical considerations

Ethical approval was obtained from the Committee of Research Ethics, Deanship of Scientific Research, Qassim University with approval number 21-07-07, following which data were collected. The participants were informed about the purpose of the research, and their informed consent was obtained online before answering the questions. Participation was voluntary. Ethical considerations were taken regarding the confidentiality and privacy of the collected data, and the data were only to be used for the purpose of the research. No interventions are planned in this study, and there is no conflict of interest to be disclosed. 

## Results

Data were collected from 1386 women who agreed to participate in this study. Of the total, 484 women (34.9%) reported that their menstruation had stopped as it was at least absent for 12 months. They will be mentioned in this study as post-menopausal women, and the 902 women who reported that their menstruation had not stopped (65.1%) will be referred to as pre-menopausal women. Considering the age, it was found that 36.7% of the sample was younger than 40 years of age, with there being a significant difference between the two groups (p = 0.000). Moreover, 96.9% of the sample were Saudi Arabian and 69.5% were married. Regarding educational level, most of the participants were university students or graduates, with there being a significant difference between the two groups as pre-menopausal women had a higher level of education. Further, 29.1% of women reported performing some type of exercise, in which the majority of them were pre-menopausal women. In general, 86.1% of women reported having their first menstrual cycle at 11-15 years old, and no difference was noted between the two groups. Moreover, 41.4% of women reported having more than five children, with a significant difference observed between the two groups. Furthermore, 33.6% of the women reported not using contraceptives. In addition, 16.1% of the women reported having a family history of BC, where 60.5% of them disclosed that the case is a first-degree relative (Table 1).

**Table 1:** The sociodemographic factors, menstrual history, and breast cancer history of the participants

		Total sample		Has your menstruation stopped? “Menopaused is considered if menstruation is absent for 12 months”				
				Yes		No		
		N	%	N	%	N	%	
Age (years)	<40	509	36.7%	152	31.4%	357	39.6%	0.000
	40-45	229	16.5%	77	15.9%	152	16.9%	
	46-50	218	15.7%	73	15.1%	145	16.1%	
	51-55	231	16.7%	85	17.6%	146	16.2%	
	56-60	134	9.7%	70	14.5%	64	7.1%	
	>60	65	4.7%	27	5.6%	38	4.2%	
Nationality	Saudi	1343	96.9%	469	96.9%	874	96.9%	0.996
	Non-Saudi	43	3.1%	15	3.1%	28	3.1%	
Marital status	Single	292	21.1%	19	3.9%	273	30.3%	0.000
	Married	963	69.5%	370	76.4%	593	65.7%	
	Widow	78	5.6%	64	13.2%	14	1.6%	
	Divorced	53	3.8%	31	6.4%	22	2.4%	
Educational level	Literacy	16	1.2%	15	3.1%	1	0.1%	0.000
	Primary	40	2.9%	32	6.6%	8	0.9%	
	Secondary	325	23.4%	141	29.1%	184	20.4%	
	University	946	68.3%	263	54.3%	683	75.7%	
	Other	59	4.3%	33	6.8%	26	2.9%	
Monthly income (SR)	<5000	518	37.4%	104	21.5%	414	45.9%	0.000
	5000-10,000	472	34.1%	218	45.0%	254	28.2%	
	11,000-20,000	341	24.6%	137	28.3%	204	22.6%	
	>20,000	55	4.0%	25	5.2%	30	3.3%	
Do you perform any kind of exercise regularly?	Yes	403	29.1%	170	35.1%	233	25.8%	0.000
	No	983	70.9%	314	64.9%	669	74.2%	
At what age did your menstruation start?	Less than 10 years old	90	6.5%	36	7.4%	54	6.0%	0.202
	11-15 years old	1193	86.1%	406	83.9%	787	87.3%	
	16-20 years old	97	7.0%	41	8.5%	56	6.2%	
	More than 20 years old	6	0.4%	1	0.2%	5	0.6%	
At what age did your menstruation stop?	46-50 years old			232	47.9%	0	0.0%	
	51-55 years old			212	43.8%	0	0.0%	
	56-60 years old			33	6.8%	0	0.0%	
	More than 60 years old			7	1.4%	0	0.0%	
How many children do you have?	0	334	24.8%	34	7.0%	310	34.4%	0.000
	1-2	166	12.0%	40	8.3%	126	14.0%	
	3-4	302	21.8%	88	18.2%	214	23.7%	
	5 or more	574	41.4%	322	66.5%	252	27.9%	
At what age did you have your first child?	Less than 20 years old	195	18.6%	110	24.2%	85	14.2%	0.000
	21-30 years old	789	75.1%	309	68.1%	480	80.4%	
	31-40 years old	65	6.2%	33	7.3%	32	5.4%	
	More than 40 years old	2	0.2%	2	0.4%	0	0.0%	
Have you ever used contraceptives?	No	466	33.6%	122	25.2%	344	38.1%	0.000
	The total period of use is less than a year	218	15.7%	90	18.6%	128	14.2%	
	The total period of use is more than one year	702	50.6%	272	56.2%	430	47.7%	
Do you have a family history of breast cancer?	No	1163	83.9%	380	78.5%	783	86.8%	0.000
	Yes	223	16.1%	104	21.5%	119	13.2%	
Is the case a first-degree relative?	No	88	39.5%	34	32.7%	54	45.4%	0.053
	Yes	135	60.5%	70	67.3%	65	54.6%	

Considering the knowledge of BC, it was found that 98.1% of the participants claimed that they had heard of BC. However, only 40.3% of the participants knew that not only females develop BC, with no significant difference between the two groups (p = 0.367). Moreover, 87.7% of the participants reportedly knew that BC could not be transmitted from one person to another by infection, and 71.1% knew that BC is the most common type of cancer among women in Saudi Arabia. Furthermore, 82.3% of the participants knew that breastfeeding reduces the risk of BC. Moreover, it was found that 48.1% of the participants knew that a lump in the breast could be due to hormonal changes, and 89.8% knew that periodic examination every 1-2 years helps in the early diagnosis of BC and reduces the death rate of patients. Furthermore, 95.5% of the participants knew that early diagnosis of BC increases the chances of obtaining better results. In general, it was found that 73.7% of the participants had adequate knowledge (Table 2).

**Table 2:** The knowledge of the participants about breast cancer

		N	%
Have you ever heard of breast cancer?	No	27	1.9%
	Yes	1359	98.1%
Do only females develop breast cancer? (Incorrect)	Incorrect/I do not know	828	59.7%
	Correct	558	40.3%
Can breast cancer be transmitted from one person to another by infection? (Incorrect)	Incorrect/I do not know	170	12.3%
	Correct	1216	87.7%
Is breast cancer the most common type of cancer among women in Saudi Arabia? (Correct)	Incorrect/I do not know	401	28.9%
	Correct	985	71.1%
Does breastfeeding reduce the risk of breast cancer? (Correct)	Incorrect/I do not know	245	17.7%
	Correct	1141	82.3%
A lump in the breast could be due to	I do not know	500	36.1%
	Hormonal changes	666	48.1%
	Old frozen milk	220	15.9%
Does periodic examination every 1-2 years help in early diagnosis of breast cancer and save patients from death? (Correct)	Incorrect/I do not know	142	10.2%
	Correct	1244	89.8%
Does early diagnosis of breast cancer increase the chances of getting better results (preserving the breast, saving the patient’s life)? (Correct)	Incorrect/I do not know	63	4.5%
	Correct	1323	95.5%
Knowledge level	Inadequate	364	26.3%
	Adequate	1022	73.7%

**Figure 1:**
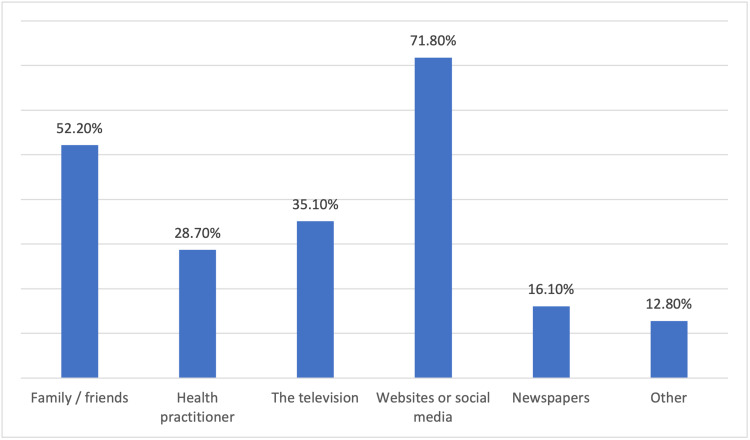
The source of knowledge regarding breast cancer for the participants

In terms of the source of knowledge of the participants regarding BC, websites or social media is considered the main source for 71.8% of the participants, followed by family and friends (52.2%) and television (35.1%), while health practitioners were the source of knowledge for only 28.7% of the participants (Figure 1).

**Figure 2:**
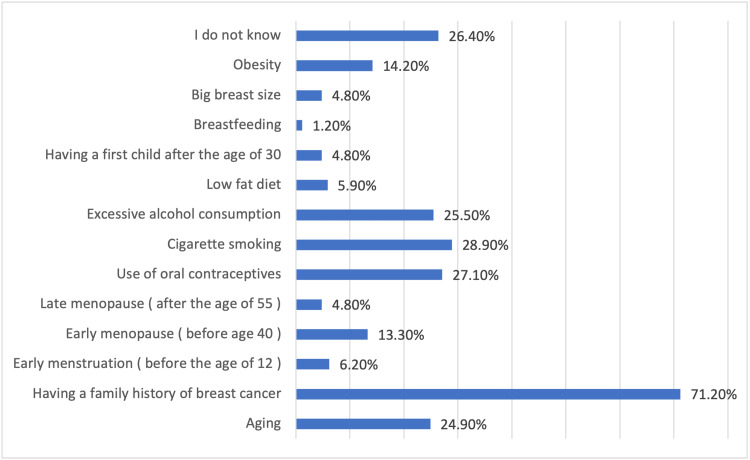
The knowledge of the participants about the risk factors associated with breast cancer

Concerning the knowledge about the risk factors of BC, it was found that 26.4% of the participants did not know them, while the main risk factors known by the participants were having a family history of BC (71.2%), smoking (28.9%), use of oral contraceptives (27.1%), and excessive alcohol consumption (25.5%) (Figure 2).

**Figure 3:**
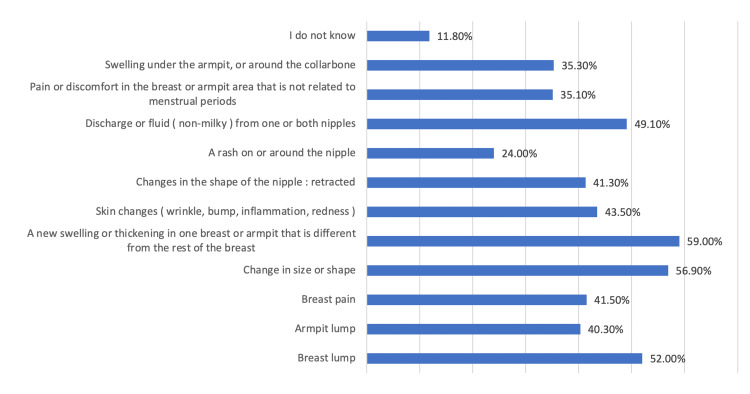
The knowledge of the participants about breast cancer symptoms

Moreover, 11.8% of the participants did not know any of the symptoms of BC, while the main symptoms known by the participants were having new swelling in one breast or armpit that is different from the rest of the breast (59%), change in size or shape (56.9%), breast lump (52.0%), discharge or fluid from one or both nipples (49.1%), skin changes (43.5%), and changes in the shape of the nipple (41.3%) (Figure 3). 

Moreover, 76.3% of the participants correctly identified radiographic examination (mammogram) as a method of screening, followed by clinical examination; however, 12.6% did not know that mammography screening and clinical examination represent the main methods of diagnosis of BC. Among the participants, 35.1% reported having undergone a clinical breast exam before. The main motivation for undergoing clinical breast examination came from suggestions from health practitioners (34.0%) and doing regular medical check-ups (31.5%), while reasons for not undergoing this test included a lack of time (46%) and not knowing why this test should be done (25.8%). Furthermore, 28.9% of the participants reported undergoing mammography before mainly as a regular check-up or because of medical advice. However, 28.4% of them did not know why this test should be done, and 17.1% had not heard about it before. Furthermore, 53.1% of the participants knew that mammography should be routinely conducted after the age of 40 even when no symptoms are present. Moreover, 16.0% of the participants reported that they had some reasons that prevented them from visiting a doctor for a breast examination; these reasons mainly included having no obvious symptoms (27.8%), fear of the results (14.0%), and embarrassment in visiting the doctor (10.3%) (Table 3).

**Table 3:** The knowledge and attitude regarding screening of breast cancer

		N	%
Methods of screening	Clinical breast examination by a doctor	943	68.0%
	Radiographic examination (mammogram)	1058	76.3%
	I do not know	174	12.6%
Methods of diagnosis	Clinical breast examination	879	63.4%
	Mammography screening (mammogram)	1018	73.4%
	Ultrasound examination	524	37.8%
	Breast biopsy	720	51.9%
	I do not know	178	12.8%
Have you ever had a clinical breast exam?	Yes	486	35.1%
	No	900	64.9%
If your answer to the previous question is yes, why?	I got advice from a health practitioner.	165	34.0%
	I noticed a lump in my breast	119	24.5%
	I have a family member with cancer	49	10.1%
	I do medical check-ups regularly	153	31.5%
If your answer is no, why?	My age group does not require an examination	183	20.3%
	I am aware, but I haven’t done it or I don’t have the time	414	46.0%
	I’ve never heard of a clinical examination before	71	7.9%
	I don’t know why I have to do this	232	25.8%
Have you ever had undergone mammography (compression chest x-ray)?	Yes	401	28.9%
	No	985	71.1%
If your answer to the previous question is yes, why?	I got advice from a health practitioner	129	32.2%
	I noticed a lump in my breast	100	24.9%
	I have a family member with cancer	42	10.5%
	I do medical check-ups regularly	130	32.4%
If your answer is no, why?	My age group does not require an examination	207	21.0%
	I am aware, but I haven’t done it or I don’t have the time	330	33.5%
	I’ve never heard of a clinical examination before	168	17.1%
	I don’t know why I have to do this	280	28.4%
When should mammography be done?	When I have a breast problem (itching, pain, lump, etc.)	293	21.1%
	At the request of the doctor	357	25.8%
	Routinely after age 40, even when there are no symptoms	736	53.1%
Do you have reasons that prevent you from visiting a doctor for a breast examination?	Yes	222	16.0%
	No	1164	84.0%

**Figure 4:**
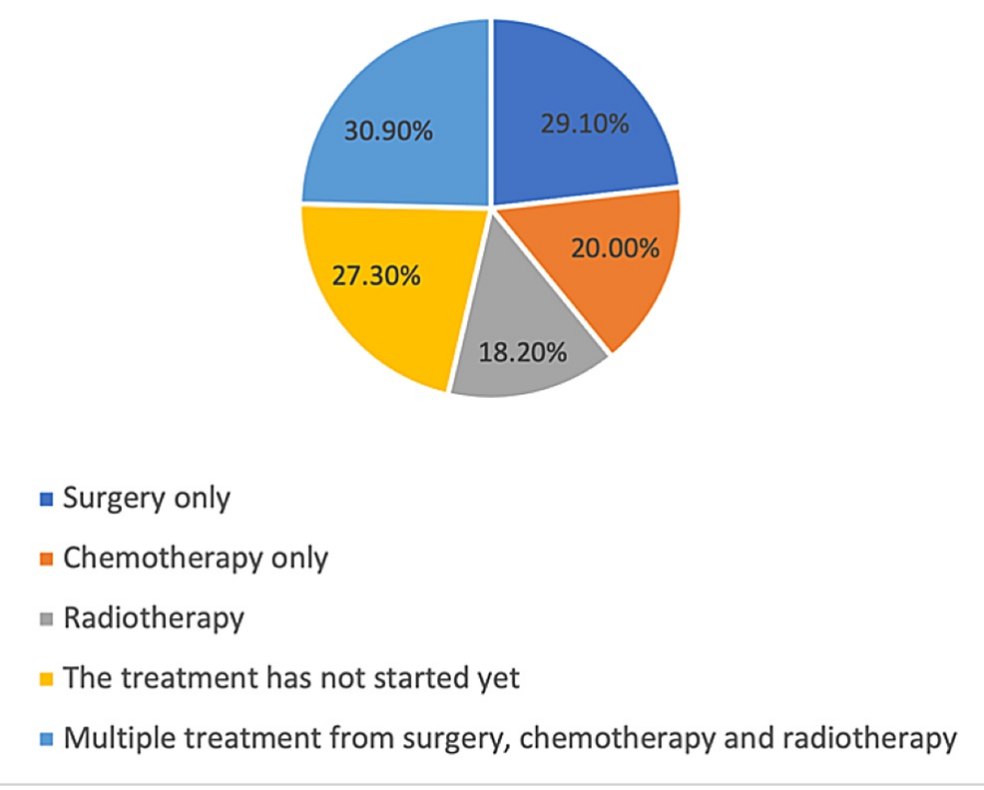
Treatment strategies of patients with breast cancer

Among the participants, the prevalence of BC was 4.9%, where 43.5% of them were diagnosed because of having symptoms or physical change and went to see a doctor, and 29% were diagnosed through mammography, as part of a preventive BC screening program. Moreover, 27.9% of the participants had no symptoms at the time of diagnosis, while 16.2% suffered from the symptoms for 2-5 months before going to the doctor. Furthermore, 27.9% had to wait for one week for their first appointment with a doctor, and 14.7% had to wait for more than five weeks; 65.2% of the participants attended 1-3 sessions with the doctor before they were diagnosed with BC (Table 4). The main symptoms associated with patients with BC include having a lump, bulge, or thickening of tissue in the breast or armpit (50.0%) and a change in the appearance of breasts (32.4%). Considering treatment among patients with BC, it was found that 30.9% of the patients needed multiple treatments involving surgery, chemotherapy, and radiotherapy, while only surgery was needed for 29.10% (Figure 4).

**Table 4:** The prevalence of breast cancer and the characteristics of patients with breast cancer

		N	%
Have you ever been diagnosed with breast cancer?	Yes	68	4.9%
	No	1318	95.1%
Which of the following best describes the events that led to your breast cancer diagnosis?	I had symptoms/I noticed a physical change and went to see a doctor (e.g., a GP)	30	43.5%
	I had symptoms/I noticed a physical change and went/was taken to the emergency room	4	5.8%
	I was seeing the doctor and had symptoms, but I went/was taken to the ER when things got worse	4	5.8%
	I was undergoing tests for another problem, during which time the cancer was discovered	6	8.7%
	I underwent mammography (compression chest x-ray ), as part of a preventive breast cancer screening program	20	29.0%
	Other	5	7.2%
Approximately, how long did you suffer from health problems or symptoms before going to the doctor for the first time?	Less than a week	4	5.9%
	1-2 weeks	7	10.3%
	3-4 weeks	10	14.7%
	5-7 weeks	5	7.4%
	2-5 months	11	16.2%
	6-12 months	3	4.4%
	More than 12 months	9	13.2%
	I had no health problems or symptoms	19	27.9%
How long did it take for you to get your first appointment with the doctor to discuss your health concerns and symptoms?	On the same day/the next day	18	26.5%
	Within one week	19	27.9%
	1-2 weeks	14	20.6%
	3-4 weeks	7	10.3%
	More than 5 weeks	10	14.7%
How often did you visit the following to get your symptoms checked before you were diagnosed with cancer? (general physician, specialist, consultant)	1-3	45	65.2%
	4-6	14	20.2%
	7-9	4	5.8%
	>10	6	8.7%

In this study, a significant difference was found between pre-menopausal and post-menopausal women concerning knowledge of BC (p = 0.042) as pre-menopausal women showed a higher level of knowledge (75.5% had adequate knowledge compared with 70.5% of post-menopausal women). Age did not prove to be a significant factor affecting the level of knowledge (p = 0.233). Moreover, it was found that marital status has a significant effect on the participant's level of knowledge; widowed participants had the lowest level of knowledge, followed by single participants, while married participants demonstrated the highest level of knowledge of BC (p = 0.000). Furthermore, the higher educational level of the participants was significantly associated with a higher level of knowledge (p = 0.000). On the other hand, performing exercises or having BC did not have a significant effect on the level of knowledge (p = 0.606, 0.276, respectively) (Table 5).

**Table 5:** The relation between demographic factors and knowledge level of breast cancer

		Knowledge level of breast cancer				
		Inadequate		Adequate		
		N	%	N	%	
Has your menstruation stopped?	Yes	143	29.5%	341	70.5%	0.042
	No	221	24.5%	681	75.5%	
Age (years)	<40	144	28.3%	365	71.7%	0.233
	40-45	50	21.8%	179	78.2%	
	46-50	52	23.9%	166	76.1%	
	51-55	57	24.7%	174	75.3%	
	56-60	39	29.1%	95	70.9%	
	>60	22	33.8%	43	66.2%	
Marital status	Single	83	28.4%	209	71.6%	0.000
	Married	231	24.0%	732	76.0%	
	Widow	36	46.2%	42	53.8%	
	Divorced	14	26.4%	39	73.6%	
Educational level	Literacy	7	43.8%	9	56.3%	0.000
	Primary	17	42.5%	23	57.5%	
	Secondary	109	33.5%	216	66.5%	
	University	208	22.0%	738	78.0%	
	Other	23	39.0%	36	61.0%	
Monthly income (SR)	<5000	140	27.0%	378	73.0%	0.008
	5000-10,000	143	30.3%	329	69.7%	
	11,000-20,000	67	19.6%	274	80.4%	
	>20,000	14	25.5%	41	74.5%	
Do you perform any kind of exercise regularly?	Yes	102	25.3%	301	74.7%	0.606
	No	262	26.7%	721	73.3%	
Have you ever been diagnosed with breast cancer?	Yes	14	20.6%	54	79.4%	0.276
	No	350	26.6%	968	73.4%	

## Discussion

In Saudi Arabia, BC is considered one of the most common cancers among the female population, ranking first among them and being responsible for 20.6% of all newly diagnosed cancers among women [6]. It is usually presented at advanced stages and is considered the leading cause of cancer mortality among females, accounting for 14% of all deaths due to cancer among females [9]. Knowledge and awareness of the general population play a vital role in the early detection and adequate treatment of BC [14]. Education and awareness are known to lead to more effective screening and early detection, which are associated with better management of the condition. Thus, this study was designed in order to assess the level of awareness about BC among the Saudi female population, focusing on post-menopausal women. In this study, 26.3% of the participants had inadequate knowledge concerning BC. Alsowiyan A et al. used the same tool to assess the knowledge as we did, and the authors reported that 38.9% of women had poor knowledge about BC, while only 61% had adequate knowledge [13]. This is consistent with the results from other studies including that of Habib et al., who reported that 34% of the university students had poor knowledge regarding BC [15], and that of Ahmed et al., who reported that among 254 medical students, 33.4 had poor knowledge regarding BC [16]. In another study conducted in the eastern region of Saudi Arabia, the authors reported that 29.3% of the Saudi female population had a low level of knowledge about BC [17], and a second study showed that 23.6% of female students in the central region of Saudi Arabia had low knowledge [18]. These studies show similar results to ours; however, there was a difference in the targeted population, which confirmed our results that indicated that age has no significant impact on the level of knowledge. This is not consistent with the results of some studies that found a significant difference between participants younger than 40 years and older patients [4,8,13]. The present study found that pre-menopausal women had a significantly higher level of knowledge than post-menopausal women. The last result could be explained by another finding of this study that shows that the participants’ educational level has a significant impact on knowledge, with a higher educational level being associated with having better knowledge concerning BC. In our sample, a significant difference is noted between pre-menopausal and post-menopausal women with regard to educational level; 75.7% of the pre-menopausal women had university education compared with 54.3% of the post-menopausal women, thus explaining the difference between the two groups regarding the level of knowledge. 

In terms of the sources of the knowledge of women about BC, it was found that websites and social media, followed by friends and relatives, were the main sources, while less than a third of the participants depended on health practitioners in obtaining knowledge. Different studies have shown that TV or the Internet is the most common source of information for women regarding BC [15,19,20], while other studies have demonstrated that social media, healthcare workers, and family and friends are the most common sources of knowledge for women concerning BC [21,22]. These results show that most of the knowledge acquired by women regarding BC does not come from trusted sources, and this may have affected the quality of information they obtained. 

The fact that successful BC treatment significantly relies on early diagnosis, as is the case for many other types of cancer, is frustrating. The present study found that 12.6% of the participants did not know about the accurate methods of screening for BC, and having no knowledge of these methods was the reason that prevented the women from undergoing a clinical breast exam and mammography (25.8% and 28.4%, respectively). This result is similar to the results found in the study by Dandash KF and Al-Mohaimeed [4]. 

With regard to the knowledge about the risk factors of BC, it was found that having a family history of BC (71.2%), smoking (28.9%), the use of oral contraceptives (27.1%), and excessive alcohol consumption (25.5%) were the main risk factors known by the participants. This result is consistent with the results of many previous studies [15,19,23,24]. However, in another study, women cited old age as the main risk factor of BC [17], while in the present study, aging represented a risk factor for only 24.9% of the participants. The main symptoms known by the participants were having a new swelling in one breast or armpit that is different from the rest of the breast (59%), change in size or shape (56.9%), breast lump (52.0%), discharge or fluid from one or both nipples (49.1%), skin changes (43.5%), and changes in the shape of the nipple (41.3%). Another study showed that the most reported symptoms of BC were painless breast lumps (82.2%), changes in breast size (82%), and changes in the nipple (80.8%) [8].

The present study also sought to assess the prevalence of BC among the female population, finding it to be 4.9%. The important fact in this estimate is that about a third of cancer patients were diagnosed after undergoing mammography as part of a preventive BC screening program. This shows the importance of this program in the early detection and diagnosis of BC as well as the importance of increasing the activities of such programs. 

Study limitations****

This study had some limitations including relying on a self-reported questionnaire, which could lead to some personal bias and depending on the online mean of distribution may lead to sampling bias toward younger and more educated participants, which may be associated with a better level of knowledge regarding BC reported in this study. 

## Conclusions

In conclusion, this study found the knowledge of post-menopausal women as adequate; however, it is significantly lower than that of pre-menopausal women. Educational level is a significant factor that affects the level of knowledge regarding BC risk factors, different modalities of diagnostic tools, screening, and management, which indicates the importance of increasing interest in education in our society. Increasing interest and conducting awareness campaigns regarding mammography as part of preventive BC screening programs is vital in detecting BC in earlier stages.
